# Trophic transfer of Cu, Zn, Cd, and Cr, and biomarker response for food webs in Taihu Lake, China†

**DOI:** 10.1039/c7ra11677b

**Published:** 2018-01-17

**Authors:** Jinxing Zuo, Wenhong Fan, Xiaolong Wang, Jinqian Ren, YiLin Zhang, Xiangrui Wang, Yuan Zhang, Tao Yu, Xiaomin Li

**Affiliations:** School of Space and Environment, Beihang University No. 37, XueYuan Road, HaiDian District Beijing 100191 PR China fanwh@buaa.edu.cn +86-10-82338162 +86-10-82338830; Chinese Research Academy of Environmental Sciences Beijing 100012 PR China

## Abstract

Water, sediments, and aquatic organism samples were collected from Taihu Lake in China. Four types of typical heavy metal (Cu, Zn, Cd, and Cr) were analyzed to evaluate their concentrations and trophic transfer in food webs. The stable nitrogen isotope δ^15^N was used to investigate the trophic interactions. The concentrations of Cd and Zn in the sediments of Taihu Lake exceeded Level I of the China National Quality Standards for Soil. Zn accumulation was identified to increase with the trophic level. The bioconcentration of the four heavy metals in aquatic organisms was evident, with the invertebrates showing the highest bioconcentration factor in the food webs. Several biomarkers were investigated, including metallothionein (MT), malondialdehyde, and Na^+^/K^+^-adenosine triphosphatase activity. A positive correlation relationship was found between the MT content and heavy metal accumulation in organism tissues.

## Abstract 2

Samples were analyzed from Taihu Lake, the bioconcentration of metals was evident and MTs were used to monitor the heavy metal pollution.

## Graphical abstract

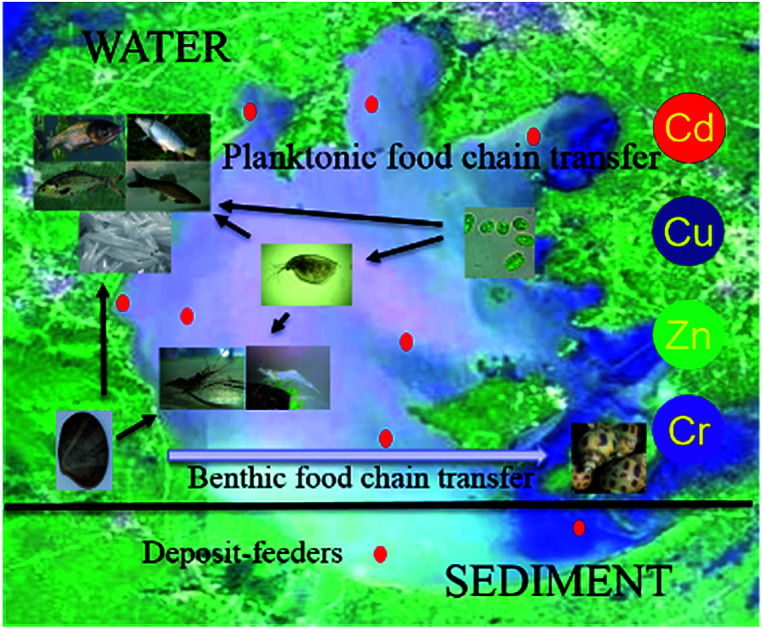

## 1. Introduction

Concern about heavy metal contamination is increasing because human activities are continuously emitting heavy metals into the environment, especially in densely populated and industrialized areas.^1^ Given the potential human health risks of heavy metals such as Cu, Zn, Cd, and Cr, their persistence, toxicity, and accumulation in the environment and the bodies of organisms are attracting considerable attention worldwide.^2,3^ China, the most populous country, has suffered from extensive heavy metal pollution hazards over the past decades with the rapid development of its economy. Increasing incidents of metal pollution have been reported. Thus, the Chinese government has placed great importance on this situation in recent years.^4–6^

Taihu Lake, located in the Yangtze Delta, is one of the most densely populated and most economically developed areas in China. It is important for limnology, regional hydrology, and for fish production. It is also an irreplaceable drinking water source for many local cities.^7^ With the rapid urbanization around the Taihu region, it receives large amounts of wastes from municipal sewage, industrial wastewater, aquaculture, *etc.*, causing serious heavy metal pollution and eutrophication. Accordingly, the Chinese scientific communities and government have paid increasing attention to the water quality safety and potential threat in this region.^8^ A comprehensive investigation on the major heavy metal elements in Taihu Lake is imperative. Most of researchers have reported on the heavy metal occurrence in Lake Taihu. However, these researchers either collected samples from limited locations of the lake, or focused only on metals in water and sediments.^9,10^

Many studies have performed the transfer of metals through the aquatic food web, and substantial progress has been made over the past decades, such as Cui *et al.* reported in newly-formed wetlands of the Yellow River Delta, China,^11^ Quinn *et al.* researched in two streams within the New World Mining District near Cooke City, Montana, USA,^12^ and Wang studied among different marine food webs.^13^ Dietary exposure is widely recognized as a major route for the transfer of metals in aquatic food webs.^14^ Currently, stable isotopes are used to investigate the trophic relationships and potential biomagnification of contaminants in food webs. δ^15^N is effective in quantifying the trophic position because the enrichment of nitrogen isotope incrementally occurs across trophic levels (TLs) with a constant rate (3–4‰).^15,16^ Furthermore, biomarkers can reflect exposure and provide a reliable indication of toxic effects. Among them, metallothioneins (MTs) in aquatic organisms has become a promising tool for detecting metal contaminations.^17,18^

The present study aimed to (1) determine the levels of heavy metals (Zn, Cu, Cd, and Cr) in food webs in Taihu Lake, (2) evaluate and quantify the biomagnification of heavy metals by calculating the bioaccumulation factor (BAF) and biomagnification factor (BMF), and (3) determine several biomarkers in organisms and assess the relationship between these biomarkers and the heavy metal content.

## 2. Study area and methods

### 2.1. Study area

**Fig. 1:**
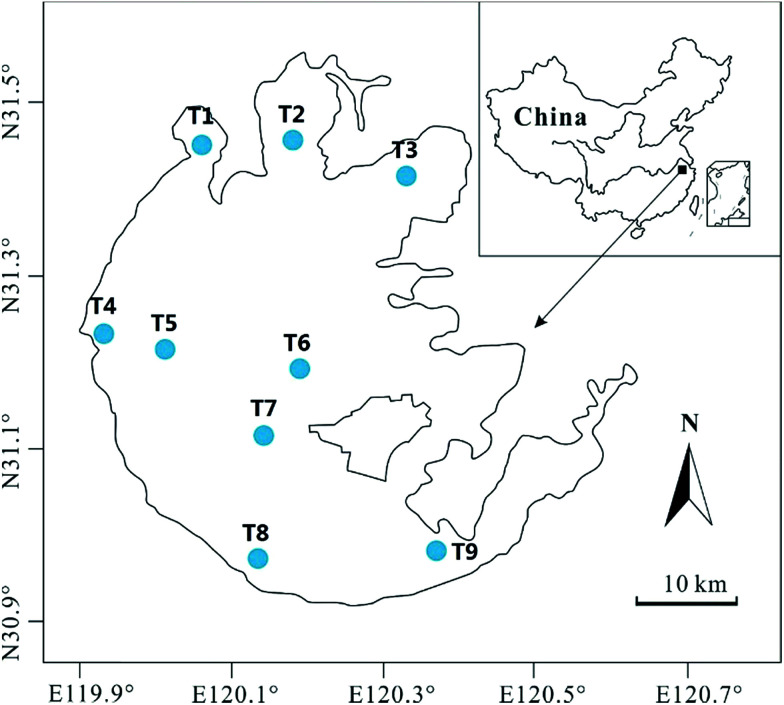
Study area with the designed sites for sample collection in Taihu Lake.

Taihu Lake is the third largest freshwater lake in east China, with an area of 2428 km^2^. It lies on the hinterland of the Yangtze Delta, spanning from 30°55′42′′ N to 31°33′50′′ N, and from 119°53′45′′ E to 126°36′15′′ E (Fig. 1). The lake is shallow, with an average water depth of only 1.9 m.^19^ Water from Taihu Lake drains into the Lou, Wusong, and Dong rivers, which discharge into the East China Sea. In recent years, Taihu Lake has accommodated large amounts of agricultural, municipal, and industrial wastewaters caused by the rapid economic development around the Taihu region. Common carp (*Cyprinus carpio*), silver carp (*Hypophthalmichthys molitrix*), whitebait (*Hemisalanx prognathous*), and bighead carp (*Aristichthys nobilis*) are the main fish species in Taihu Lake.^20^

### 2.2. Sampling

Samples, including water, sediments, plankton, invertebrate, and fish, were collected from 9 sites in different representative parts of the lake from April to May 2010 (Fig. 1). Water samples were collected at 1–1.5 m depth with a depth hydrophore, and bottom sediment samples were collected using a cylindrical corer with a diameter of 10 cm. All water column samples for metal measurement were stored in pre-cleaned polythene bottles containing a drop of 1 mol of HCl to preserve the samples, and then transported at 4 °C for laboratory analysis. The sediment samples were segmented every 5 cm from top to bottom on site. The segmented sediment cores for each sampling site were preserved in polythene bags and transported to the laboratory for subsequent processing. At each site, three parallels were collected.

Towing of the plankton conical net was performed to obtain phytoplankton (30 μm mesh) and zooplankton (270 μm mesh) samples. Shrimps and fish were collected from the area near the sampling sites using local commercial gill nets. The samples, which have been showed in Table 2, included mixed phytoplankton, mixed zooplankton, two invertebrate species, namely, white shrimp (*Exopalaemon modestus*) and freshwater shrimp (*Macrobrachium nipponense*); and six fish species, namely, anchovy (*Coilia ectenes taihuensis*), whitebait (*Hemisalanx prognathus*), white semiknife-carp (*Hemicculter leuciclus*), silver carp (*Hypophthalmichthys molitrix*), bighead carp (*Aristichthys nobilis*), and common carp (*Cyprinus carpio*). All organism samples were stored at −20 °C before analysis.

### 2.3. Analysis for heavy metals

Before the digestion, we used natural withering to dry up the sediments. And for those animals and plants, they were dehydrated at the temperature of 80 °C for one night to test their dry weight. Filtered water samples were digested with 68% HNO_3_ (Aristar grade) at 110 °C. The Cu, Zn, Cr, Cd and other HMs were then measured by ICP-MS (VG PQ2 TURBO), and the metals speciation of them was calculated with Visual MINTEQ (ver. 3.1). The sediment and organism samples were pooled and homogenized. All sediment samples were then digested using the National Standard Method (GB/T17140-1997) with HCl–HNO_3_–HF–HClO_4_ acid. The extracted solution from each sediment sample was analyzed for the total content of the four metals using a flame atomic absorbance spectrophotometer (Hitachi Z-2000). Organism samples were digested by a microwave system with HNO_3_ under controlled pressure. The concentrations of metals were determined by ICP-MS (VG PQ2 TURBO). Each sample had three replicates. The metal concentrations were calculated based on the dry weight of the sediments and organism samples. For quality assurance and quality control, standard reference materials and process blanks were digested and analyzed with each batch of samples.

### 2.4. Stable isotopes

The whole body of phytoplankton and zooplankton were adopted for stable isotope analyze. While for those invertebrates, we separated them with their crusts and tested all those tissues left. As for fishes, the muscles were used to realize isotope analyze. After drying at 50 °C (48 h), the samples were ground into a homogeneous powder and treated with a 2 : 1 chloroform : methanol solution to remove lipids. Before isotope analysis, the samples were further dried at 80 °C for 4 h, and then 0.5 mg of samples were set in 8 mm × 5 mm Sn capsules and combusted from 1000 °C to 1050 °C. Nitrogen was transported through the interface and analyzed using a mass spectrometer. Stable isotope ratios^16^ were expressed in δ notation according to the following:

1 δ^15^N = ((*R*_sample_/*R*_standard_) − 1) × 1000 (‰)

The values of *R*_standard_ can be determined based on atmospheric nitrogen.

The trophic level (TL) for each aquatic organism is given as

2 TL_consumer_ = 1.5 + (δ^15^N_consumer_ − δ^15^N_phytoplankton_)/3.8

where TL_consumer_ is the TL and the TL of phytoplankton is assumed to be 1.5.^21^

### 2.5. Analysis of MT, malondialdehyde (MDA), and Na^+^/K^+^-adenosine triphosphatase (Na^+^/K^+^-ATPase) activity

Biological samples from Dapu sampling site (T4) were chosen for measuring the concentration of biomarkers as all the aquatic organisms could be collected there. And for invertebrates, the shrimp tissues after dissecting off their crusts (using high quality stainless steel scissors on a clean glass Petri dishes) were used for measuring, while only the muscles were detected for fishes. MT measurement was performed using a modified silver saturation method.^22^ Organism samples were weighed and homogenized by ultrasonication in 0.5 mL of sucrose buffer (0.25 M sucrose, 0.1 M Tris–HCl, pH = 8.6), and centrifuged at 16 000 × *g* in a refrigerated centrifuge for 20 min. The supernatant was diluted with the homogenate to 1.5 mL, and then mixed with Ag^+^ solution. After incubation at room temperature, 0.1 mL of red blood cell hemolysate was pipetted into the mixture. The newly homogenized solution was heated in a boiling water bath (100 °C) and subsequently centrifuged. The process was repeated twice prior to the collection of the final supernatant, which was digested with HNO_3_ and diluted. The Ag concentration was measured using ICP-MS. Three replicates of biological samples from T4 site were analyzed by the same process described above. The MT content in the samples was calculated as follows:3
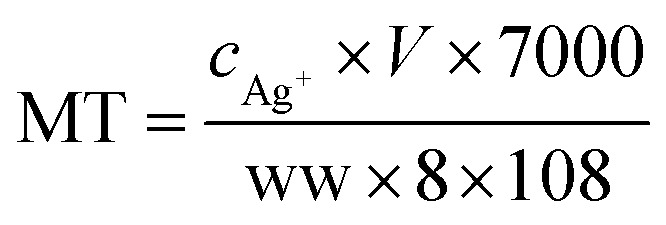
where *c*_Ag^+^_ is the Ag^+^ concentration in the final mixture, *V* is the total volume of final mixture, and ww is the wet weight of each sample.

The homogenates for MDA content and Na^+^/K^+^-ATPase activity were obtained by the methods discussed in the MT quantification. The MDA content in organism tissues were assayed using commercially available kits (Nanjing Jiancheng Bioengineering Institute, China) according to the manufacturer's protocol. The MDA concentrations were expressed as nanomole MDA per milligram protein. MDA assays were conducted using the thiobarbituric acid colorimetric method.^23^ The Na^+^/K^+^-ATPase activity was measured by determining the amount of inorganic phosphate (P_i_) liberated from the hydrolysis of the substrate ATP.^24^

### 2.6. Calculations for BMFs

The BMF was calculated by

4 BMF = (metal_predator_/metal_prey_)/(TL_predator_/TL_prey_)

where metal_predator_ and metal_prey_ are the metal concentrations of the predator and prey species, respectively.^25^

The bioaccumulation factor (BAF) is defined as

5 BAF = *C*_ss_/*C*_w_

where *C*_ss_ is the heavy metal concentration (HMC) in organisms at a steady state (mg g^−1^ DW), and *C*_w_ is the HMC in water (mg mL^−1^).^26^

### 2.7. Statistical analysis

Statistical analysis was performed using SPSS (ver 12.0, SPSS Inc., Chicago, IL, USA). Linear regression models were used to examine the associations among the HMCs and stable isotopic ratios (δ^15^N). When the *p* value was less than 0.05, the linear regression between the HMC and TL was considered significant. Half of the value of the limit of detection for each element was substituted for the values below the limit of detection, and then applied in the statistical analysis.

## 3. Results and discussion

### 3.1. HMC in environmental media and organisms

The HMCs in the environmental media and organisms are shown in Table 1. The ranges of the metal concentrations in water were in the following order (in μg L^−1^): Cd (0.005–0.033) < Cr (0.213–0.395) < Cu (1.170–3.013) < Zn (0.553–5.100). The total metal concentrations in sediments had minor variations among stations with the following ranges (μg g^−1^): Zn (68.12–158.92) > Cr (28.40–44.00) > Cu (16.75–23.20) > Cd (0.06–0.50). The range of metal concentrations in water from Taihu Lake was lower than Level I of the China National Environmental Quality Standards for Surface Water (Zn 50 μg L^−1^, Cu 10 μg L^−1^, Cd 1 μg L^−1^, Cr 10 μg L^−1^) (Level I is mainly applicable to water from natural sources and national nature reserves; the National Standard Method GB3838-2002). The Cu and Cr concentrations in sediments were in compliance with Level I of the China National Quality Standards for soil (Cu 35 μg g^−1^, Cr 90 μg g^−1^) (Level I is for protecting natural ecosystems and maintaining the soil baseline), whereas Zn and Cd were in compliance with level II of the China National Quality Standards for soil (Zn 250 μg g^−1^, Cd 0.60 μg g^−1^) (Level II is for agricultural production and human health; the National Standard Method GB15618-1995). The settlement of electroplating, printing, and dyeing wastewaters possibly led to a higher Cd concentration. Compared with previous reports, the HMCs in water and sediments were found to be declining in Taihu Lake. This finding indicated that the Chinese government and scientific community are achieving initial success in the pollution control of Taihu Lake over the past decades.^27–29^ The metal speciation of Cu, Zn and Cd in water samples showed that Cu mainly existed in Cu–DOM complexing form whereas Zn and Cd mainly existed as free ion (detailed information was listed in Table S2†).

**Table 1:** Heavy metal concentrations in water (μg L^−1^), sediment (μg g^−1^ dry weight) and organisms (μg g^−1^ dry weight) (mean ± SD, *n* = 3)

Names	Site	Cu	Zn	Cd	Cr
Water	T1	2.62	4.953	0.025	0.303
	T2	2.548	5.1	0.033	0.213
	T3	2.628	4.673	0.03	0.215
	T4	2.543	2.588	0.018	0.338
	T5	1.383	0.553	0.008	0.395
	T6	2.083	2.73	0.01	0.365
	T7	1.17	1.945	0.005	0.338
	T8	1.703	2.14	0.005	0.36
	T9	3.013	4.555	0.03	0.35
	Mean	2.188	3.248	0.018	0.319
Sediment	T1	23.2	158.92	0.22	35.4
	T2	22.56	91.16	0.32	41.68
	T3	18.15	77.46	0.06	31.9
	T4	17.87	70.64	0.32	32.84
	T5	19.54	91.56	0.09	44
	T6	16.75	68.54	0.42	30.56
	T7	20.38	69.62	0.15	33.18
	T8	18.96	68.12	0.26	30.46
	T9	17.59	78.8	0.5	28.4
	Mean	19.44	86.09	0.26	34.27
Phytoplankton		5.13 ± 0.84	22.24 ± 3.63	0.03 ± 0.01	6.01 ± 0.90
Zooplankton		3.91 ± 1.08	27.9 ± 2.95	0.02 ± 0.01	5.20 ± 0.64

**Invertebrates**					
*Exopalaemon modestus*		36.60 ± 3.86	74.17 ± 3.82	1.47 ± 0.12	6.95 ± 1.36
*Macrobrachium nipponense*		47.22 ± 4.21	65.70 ± 1.88	1.02 ± 0.36	5.41 ± 0.96
Mean		41.91	69.94	1.25	6.18

**Fish**					
*Coiliaectenes taihuensis* a		1.71 ± 0.51	55.39 ± 8.85	0.06 ± 0.01	3.10 ± 1.37
*Hemisalanx prognathus* a		1.29 ± 0.55	60.55 ± 4.52	0.23 ± 0.02	3.60 ± 0.49
*Hemicculter Leuciclus* a		1.49 ± 0.36	42.26 ± 5.73	0.06 ± 0.01	2.58 ± 0.52
*Hypophthalmichthys molitrix* a		1.74 ± 0.21	47.90 ± 7.15	0.06 ± 0.02	4.67 ± 0.76
*Hypophthalmichthys molitrix* b		4.02 ± 0.65	96.47 ± 2.19	0.05 ± 0.01	9.09 ± 2.06
*Aristichthys nobilis* a		2.62 ± 0.34	34.17 ± 3.85	0.06 ± 0.03	4.67 ± 0.76
*Aristichthys nobilis* b		4.80 ± 0.41	101.94 ± 6.36	0.17 ± 0.02	9.09 ± 2.06
*Cyprinus carpio* a		4.93 ± 0.73	132.25 ± 7.57	0.12 ± 0.05	9.32 ± 1.38
*Cyprinus carpio* b		9.74 ± 1.86	166.43 ± 1.31	0.18 ± 0.04	24.49 ± 1.75
Mean (muscles)		2.3	62.09	0.1	4.66
Mean (gill)		6.19	121.61	0.13	14.22

a Muscles of fish for HMC determination.

b Gill of fish for HMC determination.

The metal concentrations in the body of different organisms collected in Taihu Lake are also listed in Table 1. The Cu, Zn, Cr, and Cd concentrations in fish and invertebrates were similar to or lower than previous reports.^30^ Generally, the concentrations of Cu and Cd were much higher in invertebrates, whereas Cr and Zn were only slightly higher in invertebrates. Higher accumulation was found in gills than in muscles, where the adsorption of metals on to gills could be an important reason,^31^ Griffitt *et al.* also reported that gills were the primary target organ for heavy metals by histological and biochemical analysis.^32^ According to previous studies, the pathway for aquatic organisms to accumulate metals could be divided into the water and food phase.^33^ Although many studies have explored the influence of the water phase intake, the uptake of metals through the food phase may also play a significant role, as Wang suggested that the food phase exposure played a critical role to accumulate metals for most filter feeders and predators in aquatic environment.^34^ Boyle *et al.* showed the reduced reproductive output of zebrafish, which fed with polychaete (*Nereis diversicolor*) collected from a metal-impacted estuary for 68 days.^35^ In addition, De Schamphelaere *et al.* found that the growth and reproduction of *Daphnia magna* fed with contaminated algal for 21 days were obviously inhibited. However, the inhibit effects were not caused by the pathway of waterborne exposure or reduced nutritional quality of the algal.^36^ In this study, the concentration of heavy metals in water was relatively low (Table 1), so we supposed that the food phase was an important factor for aquatic organisms (except phytoplankton and other plants), which caused metals accumulation and transfer.

Interestingly, some significant variations of metals were found in different samples (Table 1). The Zn concentration was the highest in all samples, whereas the Cd concentration was the lowest. However, the Cu and Cr concentrations showed different accumulation pattern between sample groups. The Cu concentration in water and in invertebrates was higher than the Cr concentration. However, the opposite was observed in sediments, phytoplankton, zooplankton, and fish. Results showed that Cu may be more easily accumulated in the tissue of invertebrates than other organisms. This observation can be attributed to the differences in metal detoxification between the mechanisms, such as MTs, which indicate excess heavy metals.

### 3.2. TLs of organisms in the food web

The δ^15^N values and calculated TLs are presented in Table 2. The δ^15^N values ranged from 5.05 to 13.67‰, with the lowest values in the producer (phytoplankton). The δ^15^N values were converted to TLs, which increased from 1.5 to 3.8 when trophic level rose from phytoplankton to fish. However, it was found that no apparent difference occurred between phytoplankton and zooplankton as the primary consumer. It was speculated that zooplankton may also ingest particulate organic matter (POM) as main food, Wedchaparn *et al.* also reported a similar phenomenon.^38^ Besides, the planktivorous fishes exhibited rather high TL levels (*Hypophthalmichthys molitrix*, *Aristichthys nobilis*), which were even higher than omnivores. The difference of food habit may explain for that Mao *et al.* has reported that the Lake Taihu showed multiple trophic pathways, and organic matters as suspended particulate organic matter and sediment organic matter, could also serve as the bottom of the trophic chain.^39^ And they founded that the TL levels of planktivorous fish and invertebrates were higher than herbivorous fish, which was corresponding with what was observed in Table 2. The main reason should be that planktivorous fish and invertebrates could also enrich nitrogen isotope from organic matters, and such effect could be amplified considering a hypereutrophic lake Taihu Lake is.^40^ It was also interesting to find that invertebrates from Taihu Lake had abnormal higher TL levels (3.4 or 3.0), which was consistent with previous report.^41,42^ Mao *et al.* reported that macrophytes also made sizeable contribution for several invertebrate and fish species,^43^ and it was observed that consumers feed on phytodetritus or macrophytes exhibited relative higher TL levels. Therefore, it was speculated that the abnormal high calculated TL levels of invertebrates might due to their food source.

**Table 2:** The stable nitrogen isotopes (δ^15^N) and trophic level (TL) of organisms (mean ± SD, *n* = 3)

Species	Common name	Diet compositions^a^	δ^15^N‰ mean ± SD	TL
Phytoplankton	—	—	5.05 ± 0.72	1.5
Zooplankton	—	—	5.09 ± 0.42	1.5

**Invertebrates**				
*Exopalaemon modestus*	White shrimp	Zooplankton phytodetritus	12.24 ± 0.04	3.4
*Macrobrachium nipponense*	Freshwater shrimp	Meiobenthos phytodetritus	10.75 ± 0.73	3.0

**Fish**				
*Plankivores*				
*Hypophthalmichthys molitrix*	Sliver carp	Microcystis (>90%)	13.27 ± 0.02	3.7
*Aristichthys nobilis*	Terch	Microcystis (>90%)	13.35 ± 0.3	3.7

**Omnivores**				
*Coilia ectenes taihuensis*	Anchovy	Zooplankton, phytoplankton	9.14 ± 0.05	2.6
*Hemicculter leuciclus*	Black carp	Benthic animals	12.12 ± 0.84	3.4
*Cyprinus carpio*	Common carp	Macrophytes (70%), benthic animals (30%)	13.67 ± 0.14	3.8
*Hemisalanx prognathus*	Whitebait	Zooplankton (copepod, 70%; cladocera, 30%)	11.25 ± 0.43	3.1

a Diet compositions from Liu.^37^

### 3.3. Biomagnification of heavy metals

The magnification of selected heavy metals in the lake food webs was not evident, except for Zn. However, the BAF of these metals was proven to differ among various organisms (Table 3). The highest BAFs were identified in the secondary consumer. Higher BAFs of Cr and Cu were found in the producer and primary consumer, whereas a higher BAF of Zn was found in fish (Table 3). Results revealed that the primary producers had Cu and Cr concentrations above the level found in the surrounding environment. The elevated concentrations of non-essential metals (*e.g.*, Cd) in invertebrates with respect to higher TLs revealed a high availability of these metal ions or high uptake rates. Cu and Zn were also enriched, but the BAF for Zn in plankton was low, whereas the factor for Cu was lowest in fish. Cu and Zn were probably essential metals required by physiological processes, and may have different internal regulation ways in the cells of aquatic organisms.^14^

**Table 3:** The values of bioconcentration factor (BCF), and predator/prey biomagnification factor (BMF) for heavy metals in Taihu Lake

			Cd	Zn	Cu	Cr
**BAF**						
		Phytoplankton	1667	1884	10 165	16 082
		Zooplankton	1111	1342	12 751	12 257
		Invertebrate	69 444	21 533	19 154	19 373
		Fish	5556	19 116	1051	14 608

**BMF**						
	*Coilia ectenes taihuensis* a	Zooplankton^b^	1.73	1.15	0.25	0.34
		Phytoplankton^b^	1.15	1.44	0.19	0.3

a Predator.

b Prey.

BMF was determined based on the comparison of heavy metals in predators and preys (Table 3). Cd and Zn were transferred from prey to predator, whereas Cr and Cu were diluted in the food chain. This phenomenon can be characterized by the tight predator–prey relationships wherein only one or a few species of prey were consumed. Moreover, fish may change their diet with age and season.^44^

### 3.4. Biomarkers in several fish

**Fig. 2:**
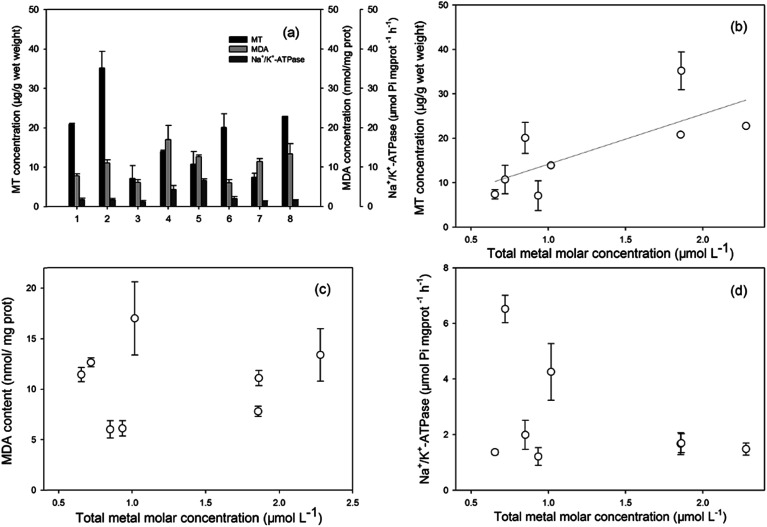
MT concentrations, MDA concentrations, and Na^+^/K^+^-ATPase activities (a) and relationship between the biomarkers ((b) MT, (c) MDA, and (d) Na^+^/K^+^-ATPase) and total metal molar concentrations in several invertebrates and fish. Each point is expressed as the mean ± standard deviation (*n* = 3).

The MT concentrations, MDA concentrations, and Na^+^/K^+^-ATPase activities, as promising biomarkers for metal pollution, oxidative stress, and biomembrane damage, respectively, were investigated. The results are presented in Fig. 2(a). The induction of MT levels indicated the exposure of invertebrates and fish to heavy metals in their habitats. The MT levels in the two invertebrate species were higher than in fish. MT functions in the regulation of essential metals, such as Cu and Zn, and in the detoxification of non-essential metals, such as Cd and Cr.^45,46^ Hence, higher accumulations of Cd and Cr in shrimps led to higher levels of MT induction, and also confirmed the highest concentration ability of heavy metals in invertebrates. For the six species of fish, MT levels were significantly higher in the three high TL species. MDA is the final product of lipid peroxidation, and one of the most damaging effects of free radicals and oxidative injury in cells.^47^ The increased MDA contents revealed that the organisms were already under a status of oxidative stress induced by some xenobiotics that probably contained heavy metals. Na^+^/K^+^-ATPases play a significant role in ionic regulation of cellular components and maintenance of tissue osmolarity, and it is also related to membrane structural integrity.^48^ In the present study, specific differences among the Na^+^/K^+^-ATPase levels in fish from Taihu Lake were not evident. The observed levels demonstrated minimal membrane damage in aquatic organisms.

The relationships between the biomarker levels and total molar concentrations of the four metals are presented in Fig. 2(b–d). Good correspondence was observed between the heavy metal accumulation levels in their tissues and the induced MTs (Fig. 2(b)). Many previous studies have indicated that MTs could evaluate the quality of heavy metal exposure and pollution stress, it was used as a suitable biomarker of metal toxicity or exposure. Bebianno *et al.* indicated that cadmium accumulated by mussels of *Mytilus galloprovincialis* was primarily associated with MT.^49^ Damiens *et al.* also reported that the larvae of Oysters from a hatchery from Normandy (English Channel) were exposed to copper or cadmium for 24 h, larvae accumulated copper and cadmium with an increase in MT concentrations particularly with cadmium.^50^ Thus, MTs in aquatic organisms could be a tool for the biomonitoring of heavy metal pollutants in ecotoxicological studies. Regarding the MDA concentrations and Na^+^/K^+^-ATPase activities, no clear relationship was found with the molar concentration of heavy metals, which indicated that the other pollutants (such as PAHs, PCBs, or quinolones) also had affected lipid peroxidation and membrane injury.^51,52^ To establish a possible assessment method of the distribution and ecological risk of the heavy metals in Taihu Lake, more data on biomarkers (likely GSH, SOD for defense against oxidative stress) in different fish organs and integrated biomarker response analysis are required.

## 4. Conclusions

Nine sampling points from the entire Taihu Lake were selected for the survey of the heavy metals (Cu, Zn, Cd, and Cr) in water, sediment, plankton, invertebrate and fish. The concentrations of Cd and Zn in sediments exceeded Level I of the China National Quality Standards for soil (GB 15618-1995). The δ^15^N stable isotope analysis was used to describe the transference of the four heavy metals in the food web in Taihu Lake. By analyzing the relations between trophic levels and the accumulations of heavy metals, Zn accumulation was identified to increase with the trophic level. Bioconcentration and biomagnification factors for the four metals in the food web also been calculated. The invertebrates showed the highest bioconcentration factor in the food webs. Finally, the relationships between three kinds of biomarkers (MT, MDA and Na^+^/K^+^-ATPase activity) and the heavy metal accumulations was investigated. A positive correlation between MT levels and the accumulations gave us a possible tool in the passive monitoring of heavy metals pollutants in ecotoxicological studies. In summary, a comprehensive status of heavy metals is analyzed in aquatic organisms of different trophic levels from Taihu Lake, which would provide a useful theoretical basis of heavy metal pollution control and regulation for local governments.

## Compliance with ethical standards

This research did not involve human participants and the authors declare that any research involving human participants and/or animals in this study was carried out in strict accordance with the recommendations of the approval of Institutional Authority for Laboratory Animal Care of Beihang University. All efforts were made to minimize suffering of the animals.

## Conflicts of interest

The authors declare that they have no conflict of interest.

## Supplementary Material

RA-008-C7RA11677B-s001
