# Supplementary material for: Trophic transfer of Cu, Zn, Cd, and Cr, and biomarker response for food webs in Taihu Lake, China
Source: RSC Adv. 2018 Jan 17;8(7):3410–7. doi: 10.1039/c7ra11677b (PMC9077756; doi:10.1039/c7ra11677b)
Supplement: RA-008-C7RA11677B-s001 [file RA-008-C7RA11677B-s001.pdf]

Electronic Supplementary Material (ESI) for RSC Advances.
This journal is © The Royal Society of Chemistry 2018

Electronic Supplementary Material (ESI) for RSC Advances.

Trophic transfer of Cu, Zn, Cd, and Cr, and biomarker response for food webs in Taihu Lake, China

Jinxing Zuo[a], Wenhong Fan[a,]*, Xiaolong Wang[a], Jinqian Ren[a], YiLin Zhang[a], Xiangrui Wang[a], Yuan Zhang[b], Tao Yu[b], Xiaomin Li[a]

[a] *School of Space and Environment, Beihang University, Beijing 100191, PR China.*

[b] *Chinese Research Academy of Environmental Sciences, Beijing 100012, PR China.*

*Corresponding to:

Email: fanwh@buaa.edu.cn Tel: (86)-10-82338830

Supplementary Materials and Methods

Water samples were collected from 9 sites in different representative parts of Taihu Lake. The temperature and pH of the water samples were measured at the sampling sites by portable WTW VARIO portable pH/temperature analyzer, German. After infiltration through a 0.45 μm membrane (Mixed cellulose ester membrane, Whatman), the Cu, Zn, Cr, Cd and other HMs were then measured by ICP-MS (VG PQ2 TURBO), the K, Ca, Na and Mg concentrations were analyzed using ICP-OES (Optima 5300 DV), anions were measured using ion chromatography (Metrohm 792), and total organic carbon was analysised by total organic carbon analyzer (TOC-V CPH, SHIMADZU). Each sample had three replicates. The metals speciation of Cu, Zn, Cr, and Cd in filtered water samples was simulated and analyzed with Visual MINTEQ (ver. 3.1).

Supplementary Results

Table S1 Main water quality parameters of Taihu Lake.

sample	TOC (ppm)	pH	T (℃)	K (ppm)	Na (ppm)	Ca (ppm)	Mg (ppm)	CL^- (ppm)	SO_4^{2-} (ppm)	NO_3^- (ppm)	CO_3^{2-} (ppm)
T1	25.91	7.58	22.1	5.34	48.8	37	10.1	50.06	64.8	4.56	72.1
T2	25.27	6.88	21.5	5.62	27.5	33.6	7.81	24.11	21.3	3.2	87
T3	31.25	7.86	22.2	5.69	57.9	32.8	4.28	16.5	23.6	4.25	75.2
T4	29.54	6.65	20.8	4.4	45.2	34	9.32	31.51	18.8	3.86	73.9
T5	25.89	6.54	20.6	3.74	61.4	39.1	7.81	40.78	24.4	1.2	76.7
T6	22.85	6.54	19.8	3.83	9.02	26.5	5.04	9.98	11	2.59	67.4
T7	22.27	6.68	20.6	3.75	23.6	34.4	7.81	31.51	18.8	0.88	69.3
T8	20.69	6.75	21.2	4.02	12.1	26.98	6.85	27.42	23.4	1.36	65.4
T9	21.25	6.86	19.8	3.68	28.32	1.25	5.89	36.85	34.2	1.89	58.2
mean	24.99	6.93	21.5	4.45	34.87	29.51	7.21	29.86	26.70	2.64	71.69

Table S2 The metal speciation of Cu, Zn, Cr and Cd in water samples of Taihu Lake.(%)

Metal		T1	T2	T3	T4	T5	T6	T7	T8	T9	Mean
Cd	Cd^{2+}	69.906	75.189	69.277	73.339	75.433	76.485	76.933	75.112	76.648	69.813
	$Cd\text{-}OH^-$	0.114	0.025	0.222	0.014	0.011	0.012	0.017	0.025	0.012	0.050
	Cd-DOM	14.878	16.989	20.342	18.998	15.162	19.43	14.909	16.675	17.940	21.702
	$Cd\text{-}Cl^-$	6.716	3.648	2.279	4.605	6.021	1.624	4.921	4.268	2.864	4.105
	$Cd\text{-}SO_4^{2-}$	4.812	1.877	1.908	1.568	2.004	1.123	1.707	2.209	1.768	2.108
	$Cd\text{-}NO_3^-$	0.014	0.011	0.013	0.012	-	-	-	-	-	0.012
	$Cd\text{-}CO_3^{2-}$	3.559	2.247	5.947	1.459	1.358	1.312	1.507	1.699	0.757	2.205
Cr	CrO_4^{2-}	75.998	62.503	78.089	54.599	49.481	49.866	55.104	63.232	72.286	62.351
	$HCrO_4^-$	4.993	21.398	2.78	31.451	36.212	38.915	30.019	23.113	26.844	23.969
	$NaCrO_4^-$	0.576	0.282	0.734	0.4	0.483	0.078	0.214	0.129	0.351	0.361
	$KCrO_4^-$	0.028	0.025	0.032	0.017	0.013	0.015	0.015	0.019	0.020	0.020
	$CaCrO_4^-$	18.404	15.792	18.365	13.533	13.810	11.127	14.648	13.508	0.499	13.298
Cu	Cu^{2+}	9.265	8.546	3.379	8.274	10.436	8.569	10.494	9.408	1.536	7.767
	$Cu\text{-}OH^-$	1.321	1.325	5.337	0.752	0.716	0.627	1.021	0.931	0.233	1.363
	Cu-DOM	78.314	76.853	79.29	85.302	83.496	86.638	80.951	83.04	96.574	83.384
	$Cu\text{-}Cl^-$	0.018	-	-	0.010	0.017	-	0.013	0.011	-	0.014
	$Cu\text{-}SO_4^{2-}$	0.621	0.209	0.291	0.010	0.271	0.123	0.228	0.271	0.072	0.233
	$Cu\text{-}NO_3^-$	-	-	-	-	-	-	-	-	-	-
	$Cu\text{-}CO_3^{2-}$	10.450	13.058	11.698	5.491	5.059	4.034	7.282	6.33	1.578	7.220
Zn	Zn^{2+}	65.336	70.102	56.941	68.542	73.057	69.490	73.724	70.669	67.920	68.420
	$Zn\text{-}OH^-$	2.201	0.336	5.154	0.182	0.138	0.140	0.214	0.328	0.132	0.981
	Zn-DOM	22.034	25.099	26.494	28.137	23.273	27.974	22.642	24.865	29.863	25.598
	$Zn\text{-}Cl^-$	0.184	0.100	0.055	0.127	0.171	0.044	0.139	0.118	0.064	0.111

Metal	T1	T2	T3	T4	T5	T6	T7	T8	T9	Mean
$Zn\text{-}SO_4^{2-}$	4.256	1.662	1.489	1.391	1.843	0.969	1.553	1.973	1.279	1.824
$Zn\text{-}NO_3^{-}$	-	-	-	-	-	-	-	-	-	-
$Zn\text{-}CO_3^{2-}$	5.974	2.691	9.855	1.61	1.503	1.367	1.723	2.04	0.736	3.055

NOTE: “-“ means no simulation results
